# Investigating transcriptional regulation of *Plasmodium falciparum* upon drug perturbation

**DOI:** 10.1186/1475-2875-9-S2-O32

**Published:** 2010-10-20

**Authors:** Tharina van Brummlen, John VW Becker, Dalu T Mancama, Heinrich Hoppe

**Affiliations:** 1 CSIR, Biosciences, PO Box 395, Pretoria, 0001, South Africa

## 

Gene regulation of the malaria parasite *Plasmodium falciparum* has proven to be complex with evidence supporting both transcriptional [1,2] and post-transcriptional level control [3,4]. Transcriptional profiling of environmentally perturbed malaria parasites can reveal functionally related genes with common regulatory mechanisms that are responsive to external stimuli [2]. By interrogating microarray data derived from *P. falciparum* populations treated with several drug classes, a subset of four genes potentially involved with the parasite's survival mechanisms following drug perturbation were identified, i.e. general stress response genes. In addition, data from two independent functional genomics investigations on the response of *P. falciparum* parasites to polyamine biosynthesis inhibitors (DFMO/MDL73811 co-inhibition [5] and cyclohexylamine [6]), revealed four genes of which the transcript abundance appear to be uniquely affected upon polyamine depletion only [5,6], i.e. perturbation-specific stress response genes. The regulation of this generalized versus perturbation-specific transcriptional responses was subsequently investigated.

In order to investigate transcriptional regulation of *P. falciparum,* stable transfection methodology was established through the use of green fluorescent protein and luciferase as transgene expression markers [7]. Subsequently, the regions 1500 bp upstream of the coding sequence (presumably containing the promoters) of the eight genes were inserted into the transfection vector driving luciferase expression, with the heat shock protein 86 promoter as control. Parasite transfection was performed and transfectants were selected for resistance against the antifolate WR99210.

Stable lines of the *P. falciparum* transfectants were exposed to a variety of compounds from different antimalarial drug classes including two polyamine biosynthesis inhibitors (i.e. artemisinin, mefloquine,doxycycline, DFMO and cyclohexylamine) and luciferase activity was quantified as a measure of promoter activity. The altered luciferase activity was used to determine whether the observed transcriptional response upon drug perturbation were indeed the result of true transcriptional control at the promoter level as opposed to alteration of mRNA stability or other post-transcriptional means.
